# Interaction between Movement Proteins of *Hibiscus green spot virus*

**DOI:** 10.3390/v14122742

**Published:** 2022-12-08

**Authors:** Anastasia K. Atabekova, Ekaterina A. Lazareva, Alexander A. Lezzhov, Anna D. Solovieva, Sergei A. Golyshev, Boris I. Skulachev, Ilya D. Solovyev, Alexander P. Savitsky, Manfred Heinlein, Sergey Y. Morozov, Andrey G. Solovyev

**Affiliations:** 1 A. N. Belozersky Institute of Physico-Chemical Biology, Moscow State University, 119992 Moscow, Russia; 2 Department of Virology, Biological Faculty, Moscow State University, 119234 Moscow, Russia; 3 A. N. Bach Institute of Biochemistry, Research Center of Biotechnology of the Russian Academy of Sciences, 119071 Moscow, Russia; 4 Institute for Plant Molecular Biology (IBMP-CNRS), University of Strasbourg, 67000 Strasbourg, France; 5 All-Russia Research Institute of Agricultural Biotechnology, 127550 Moscow, Russia

**Keywords:** plant virus, virus transport, cell-to-cell movement, movement protein, plasmodesmata, protein-protein interaction, higrevirus, *Hibiscus green spot virus*

## Abstract

Movement proteins (MPs) of plant viruses enable the translocation of viral genomes from infected to healthy cells through plasmodesmata (PD). The MPs functions involve the increase of the PD permeability and routing of viral genome both to the PD entrance and through the modified PD. *Hibiscus green spot virus* encodes two MPs, termed BMB1 and BMB2, which act in concert to accomplish virus cell-to-cell transport. BMB1, representing an NTPase/helicase domain-containing RNA-binding protein, localizes to the cytoplasm and the nucleoplasm. BMB2 is a small hydrophobic protein that interacts with the endoplasmic reticulum (ER) membranes and induces local constrictions of the ER tubules. In plant cells, BMB2 localizes to PD-associated membrane bodies (PAMBs) consisting of modified ER tubules and directs BMB1 to PAMBs. Here, we demonstrate that BMB1 and BMB2 interact in vitro and in vivo, and that their specific interaction is essential for BMB2-directed targeting of BMB1 to PAMBs. Using mutagenesis, we show that the interaction involves the C-terminal BMB1 region and the N-terminal region of BMB2.

## 1. Introduction

Plant viruses typically encode ‘movement proteins’ (MPs) enabling transport of virus genomes from infected cells to adjoining healthy cells through plasmodesmata (PD), channels interconnecting cells in plant tissues [1,2]. MPs interact with PD to increase the PD size exclusion limit, thereby increasing the PD permeability. Such functional PD modification allows the translocation of virus genomic nucleic acid through the dilated PD microchannels [2,3,4,5]. Another essential MP function is routing progeny virus genomes destined for transport out of infected cells to the PD entrance. Additionally, viral MPs are often capable of cell-to-cell transport on their own and, therefore, are believed to take part in the genome transfer through modified PDs [1,6,7]. According to current views, viral membrane-associated replication compartments (VRCs) rather than individual genomes are delivered to the PD orifice with the aid of MPs and, therefore, the progeny virus genomes produced in the PD-associated VRCs can be translocated directly to the PD channels [1,5,8]. Many MPs characterized so far have an RNA-binding activity, which can play a role in both the targeting of viral RNA genome to PD and its translocation through the PD channels [7,9].

Transport systems encoded by viruses of distant groups vary considerably in the number of MPs they comprise, in the MP properties, and the mechanisms of MP-mediated virus transport [1,10]. Single MP-based transport systems are exemplified by that of *Tobacco mosaic virus* (TMV), which codes for the 30-kDa MP capable of PD modification, cell-to-cell transport, RNA binding, and directing VRCs to PD [1,11]. More complicated transport systems can include two, three or more virus-encoded MPs. In such multicomponent transport systems, the typical MP functions, as those found for the TMV MP, are distributed among different proteins showing therefore a division of labor. On the other hand, the MPs encoded by a given virus genome act in concert, implying that these MPs interact with each other to perform the movement function. Analysis of interactions between MPs that make up multicomponent transport systems is essential for understanding the mechanisms of virus movement; however, such interactions are currently characterized only for a limited number of viruses.

In virus families *Alphaflexiviridae,* Betaflexiviridae, Virgaviridae, and Benyviridae, positive-stranded RNA genomes contain three overlapping genes of the so-called ‘triple gene block’ (TGB) coding for MPs representing a three-component transport system. These MPs include TGB1, an RNA-binding protein containing a NTPase/helicase domain, and small membrane-associated proteins termed TGB2 and TGB3 [12]. The TGB3 protein contains a signal for PD targeting, localizes to PD-associated membrane bodies (PAMBs), and enables relocalization of TGB2, which resides in the endoplasmic reticulum (ER) when expressed alone, to TGB3-containing PAMBs [13,14,15,16,17,18]. The TGB1 protein has mostly cytoplasmic localization, but in the presence of both TGB2 and TGB3 it is directed to PAMBs, the PD channels, and neighboring cells [19,20,21]. These observations imply that the TGB proteins interact in plant cells. In fact, as demonstrated for *Barley stripe mosaic virus* (BSMV), specific amino acid residues in the central hydrophilic loops located between two transmembrane segments in both TGB2 and TGB3 are required for the interaction between the two proteins, whereas TGB3 can bind TGB1 [22]. These interactions apparently account for the observed relocalization events and are essential for the TGB-mediated virus transport [22]. Studies of TGB-encoding *Bamboo mosaic virus* (BaMV) generally support the BSMV data. The interaction between BaMV TGB2 and TGB3 has been demonstrated, and a TGB2-TGB3-containing membrane-embedded complex is required for the targeting of TGB1 to PAMBs [13,21], whereby the latter depends on cysteine residues in the TGB2 C-terminal region [23]. As TGB1 interacts with TGB3 in BSMV and TGB2 in BaMV, these findings suggest that the TGB1 proteins of different viruses may interact with either component of the TGB2/TGB3 complex formed in ER-derived membranes for PD targeting [24].

The genome of *Hibiscus green spot virus* (HGSV; genus *Higrevirus*, family *Kitaviridae*) infecting *Citrus* plants has two MP genes that constitute a ‘binary movement block’ (BMB), which is evolutionary distantly related to the TGB [25,26,27,28]. The BMB1 protein has the NTPase/helicase domain similar to that of TGB1 proteins, whereas BMB2 is similar to TGB2 by carrying two transmembrane regions and showing a marginal sequence similarity of its central hydrophilic region to that in TGB2 [28]. BMB2 is localized to PAMBs and the PD interior [27,29]. Moreover, BMB2 is able to increase the PD SEL, and this BMB2 property correlates with its ability to induce constrictions of the ER tubules [29]. BMB1 is found in the cytoplasm and nucleoplasm when expressed alone and is directed to PAMBs, the PD channels and neighboring cells in the presence of BMB2 [29]. Therefore, BMB2 is functionally equivalent to the TGB2/TGB3 complex. Apparently, the ability of BMB2 to recruit BMB1 to the sites of its own location might suggest that these two proteins interact.

In this paper, the interaction between HGSV BMB1 and BMB2 is demonstrated. Using mutational analysis, the regions involved in the interaction are mapped to the C-terminal region of BMB1 and to the N-terminal region of BMB2.

## 2. Materials and Methods

### 2.1. Plasmid Construction

The recombinant constructs for the transient expression of BMB1 and BMB2 along with their N- and C-terminal fusions to fluorescent proteins, ER-mRFP, mRFP, and PVX-POL-GFP, were described previously [27,30]. For dihydrofolate reductase (DHFR) production in *Escherichia coli*, the expression vector pQE-40 (Qiagen, Hilden, Germany) was used. Primers used to generate other recombinant clones are listed in Supplementary Table S1.

To obtain pLH-GFP-BMB1d22, a portion of BMB1 gene coding for the protein C-terminal fragment lacking 22 C-terminal amino acid residues was amplified with primers BMB1C-BglII-P and BMB1C-d22-M. The resulting product was digested with restriction endonucleases *Bgl*II-*Xba*I and cloned into similarly digested pLH-GFP-BMB1 to replace the respective wild type sequence. To obtain pLH-BMB1d22, pLH-GFP-BMB1d22 was digested with *Nco*I to cut out the GFP coding sequence and ligated. To generate the RFP-fused Golgi marker, the previously described construct pLH-ST-YFP [16] was modified to replace the YFP coding sequence with that of tagRFP (Evrogen, Moscow, Russia). To obtain pLH-GFP+22, the coding region corresponding to 22 C-terminal amino acids of BMB1 was amplified on the template of pLH-GFP-BMB1 with primers BMB2-C22-P and Right. The resulting product was treated with restriction endonucleases *Bam*HI–*Xba*I and inserted into the previously described pLH-GFP-BMB2 construct to replace the BMB2 coding sequence. To obtain pLH-BMB1-BMB2, the mRFP coding sequence in the previously described construct pLH-mRFP-BMB2 was replaced by the BMB1 coding sequence as an *Xho*I-*Bam*HI fragment. To generate pLH-GFP-BMB1-BMB2, pLH-GFP-BMB1 construct was digested with *Xho*I-*Bgl*II, and the resulting fragment was cloned into the similarly digested construct pLH-BMB1-BMB2. To obtain pLH-BMB2-mN and pLH-BMB2-mN-mRFP, the coding sequences were amplified using previously described pLH-BMB2 and pLH-BMB2-mRFP as template and using BMB2-mN-P and Right primers. The resulting PCR-products were digested with *Xho*I-*Xba*I and cloned into the similarly digested binary vector pLH* [31]. To obtain pLH-BMB2-mHydr and pLH-BMB2-mHydr-mRFP, the coding sequences were amplified on the previously described pLH-BMB2 and pLH-BMB2-mRFP respectively, using primers BMB2-mHydr-P and Right. The resulting PCR-products were digested with *Xho*I-*Xba*I and cloned into the similarly digested binary vector pLH*. Overlap PCR was used to introduce mutations in the central region of BMB2. In the first step, one PCR product was obtained by using previously described pLH-BMB2 as template and Left and BMB2-mMid-ovl-M as primers, and another PCR product was obtained from pLH-BMB2 or pLH-BMB2-mRFP using primers Right and BMB2-mMid-ovl-P. In the second step, the two PCR products were fused by amplification with primers Left and Right. The resulting DNA fragments were digested with *Xho*I-*Xba*I and cloned into similarly digested binary vector pLH*.

### 2.2. Synthetic Genes

The BMB1-encoding nucleotide sequence was optimized for protein expression in *E. coli* cells. The codon-optimized BMB1 gene and the BMB2-QTY gene were synthesized (Evrogen) and subcloned into pET-33b(+) (Novagen, Madison, WI, USA). The nucleotide sequences of the synthetic BMB1 and BMB2-QTY genes are shown in Supplementary Table S2.

### 2.3. Protein Expression in Bacteria

The *E. coli* strain BL21 cells were transformed with expression vectors, and clones with highest expression levels were selected. Selected clones were grown overnight at 37 °C in the 2YT medium in the presence of kanamycin (25 μg/mL). The overnight culture was diluted 10-fold and grown at 37 °C to optical density at 600 nm (OD_600_)  =  0.8. Protein expression was induced by addition of IPTG (final concentration 1–2 mM) for 2–4 h. Cells were pelleted at 4500× *g* for 10 min. The recombinant proteins carrying the N-terminal 6xHis tag were purified on Ni-NTA agarose in accordance with the ‘The QIAexpressionist’ (Qiagen) protein isolation protocol under denaturing conditions. Purified proteins were analyzed by SDS electrophoresis in a 15% polyacrylamide gel according to Laemmli and renatured by dialysis.

### 2.4. Western Blot Analysis

To obtain samples for protein electrophoresis, *Nicotiana benthamiana* leaves were ground to a powder in liquid nitrogen and lysed in a buffer containing three parts of Tris-HCl, pH 7.5 and one part of 4× Laemmli sample buffer (100 mM Tris-HCl pH 6.8, 100 mM β-mercaptoethanol, 10% glycerol, 4% SDS, 0.1% bromophenol blue). The samples were denatured at 95 °C for 5 min and cleared from cell debris via centrifugation. Proteins were separated by 12% polyacrylamide SDS-PAGE and transferred to a Hybond-P polyvinylidene difluoride (PVDF) membrane (GE Healthcare Bio-Sciences, Niskayuna, NY, USA). Rabbit Anti-GFP antibodies conjugated with peroxidase (Rockland, Pottstown, PA, USA) were used for protein detection. After antibody incubation, bands were observed via chemiluminescence using an ECL system (GE Healthcare Bio-Sciences).

### 2.5. Far-Western

For Far-Western blotting, recombinant proteins were separated by SDS-PAGE (approximately 1 μg of protein per gel slot) using a 15% polyacrylamide gel and transferred to a nitrocellulose membrane. After transfer, the membrane was stained with a 0.1% Ponceau S solution, photographed, and quickly washed with distilled water to remove staining. The transferred proteins were then denaturated with 50 mM Tris-HCl pH 7.5, 8 M urea and 0.05% Tween buffer for 40 min at room temperature (RT). Protein renaturation and membrane blocking were performed by membrane incubation in a 5% skimmed milk tTBS buffer (0.01 M Tris-HCl pH 7.6, 0.0675 M NaCl, 0.1% Tween-20) with 1 mM DTT and 3 mM MgCl_2_ for 2 h with buffer changes every 20–30 min and then left at 4 °C overnight. Next day, a freshly dialyzed binding protein (5 μg/mL) was added to the membrane in tTBS buffer supplemented with 1 mM DTT, 3 mM MgCl_2_ and 220 mM NaCl. The membrane was incubated for 3 h at RT, then overnight at 4 °C, and finally washed 3–5 times with tTBS buffer without supplements. The bound protein was detected by ECL immunoblotting with BMB2-QTY-specific antibodies (Almabion, Voronezh, Russia).

### 2.6. Plant Material

*N. benthamiana* plants were grown and maintained in a glasshouse or in growth chambers under standard conditions (16-h/8-h light/dark cycles, 24/20 °C day/night temperatures, and nearly 50% humidity). The 5–6-week-old plants were used for transient protein expression and movement complementation assays.

### 2.7. Plant Agroinfiltration

The binary vectors were transformed into *Agrobacterium tumefaciens* (strain C58C1) using a freeze-thaw method. Agrobacterial cultures were prepared for agroinfiltration as described previously [31]. Overnight cultures of agrobacteria were grown in Luria-Bertani (LB) medium at 28 °C with appropriative antibiotics, 10 mm 2-(*N*-morpholino)ethanesulfonic acid (MES), pH 5.5, and 20 μm acetosyringone. The cell pellet was collected by centrifugation and resuspended in infiltration medium (10 mM MES, pH 5.5, 10 mM MgCl_2_, 150 mM acetosyringone). Obtained cell suspensions were incubated at room temperature for 3 h. Before infiltration, *A. tumefaciens* suspensions were diluted to a final OD_600_ =  0.3. For cell-to-cell movement complementation assays, PVX-POL-GFP was infiltrated at OD_600_ = 0.0001 to obtain individually transformed plant cells. A 2-mL syringe without needle was used to infiltrate the abaxial surface of *N. benthamiana* leaves.

### 2.8. Confocal Microscopy and Movement Complementation Visualization

Subcellular localization of proteins was visualized at the third day post agroinfiltration (d.p.a.) in epidermal cells. Leaf discs were observed with a confocal laser scanning microscope Nikon C2plus equipped with a ×60 (1.2 NA) water immersion objective. Excitation wavelengths were 488 nm for GFP and 548 nm for mRFP. Images were acquired at 495–545 nm for GFP and at 580–640 nm for mRFP and processed using Nikon NIS Elements and ImageJ (1.47 s) software.

Movement complementation assays were observed under long-wave UV light (365 nm) using a Black-Ray B-100AP lamp (UVP, Cambridge, UK).

### 2.9. FRET-FLIM

To perform FRET-FLIM (Förster resonance energy transfer between fluorophores detected by fluorescence lifetime imaging microscopy), GFP-tagged BMB1 was transiently expressed in *N. benthamiana* leaves in the presence of the potential interacting partner BMB2, either non-tagged or fused to mRFP. FRET-FLIM measurements were performed with a LIFA frequency domain fluorescence life-time imaging system (Lambert Instruments, Roden, The Netherlands) and with a DCS-120 TCSPC confocal FLIM system (Becker and Hickl, Berlin, Germany). Independent experiments were repeated 3–10 times. In each experiment, at least three leaves were used for each pair of coexpressed proteins. Measurements were carried out in a minimum of eight regions of 3–8 epidermal cells located in different leaf areas. The raw data containing lifetime information were analyzed in Microsoft Excel to calculate average lifetime values and standard deviations. Two-tailed parametric Student’s *t*-tests were used for statistical analysis. The FRET efficiency was calculated according to the formula E = 1 − (T_DA_/T_D_), where T_DA_ is the lifetime of the donor fluorophore (GFP) in the presence of the acceptor fluorophore (mRFP) and T_D_ is the donor lifetime in the absence of acceptor.

## 3. Results

### 3.1. BMB1 and BMB2 Interact In Vitro and In Vivo

**Figure 1:**
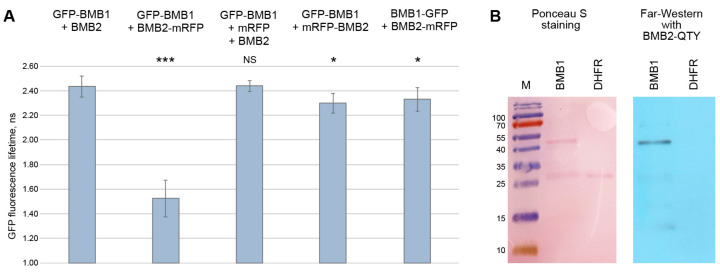
Analysis of interaction between BMB1 and BMB2. (**A**) FRET-FLIM analysis of protein interaction in cells of *N. benthamiana* leaves agroinfiltrated for expression of BMB1 and BMB2. The bar graphs represent average fluorescence lifetimes (ns), error bars indicate the standard deviation. Numbers of independent measurements (N) were as follows: GFP-BMB1 + BMB2, 283; GFP-BMB1 + BMB2-mRFP, 463; GFP-BMB1 + mRFP + BMB2, 134; GFP-BMB1 + mRFP-BMB2, 133; BMB1-GFP + BMB2-mRFP, 172. Above the bars, the significance of difference from the data for GFP-BMB1 + BMB2 is indicated; asterisks indicate a statistically significant difference (***, *p* < 0.001; *, *p* < 0.05) according to a Student’s *t*-test; NS—not statistically significant (*p* ≥ 0.05). (**B**) Far-Western analysis of interaction between the bacteria-expressed 6x-His-tagged BMB1 and BMB2-QTY, a BMB2 version lacking hydrophobic membrane-interacting regions. Left, Ponceau S staining of membrane with proteins transferred from a protein gel (BMB1, 43 kDa; DHFR, 25 kDa). Right, detection of BMB2-QTY after incubation of the membrane with a preparation of purified BMB2-QTY. M, molecular weight markers. Sizes of individual protein bands are indicated in kDa.

As BMB1 was found to be targeted to PAMBs by BMB2 [27], an interaction between BMB1 and BMB2 could be anticipated. To verify this hypothesis, FRET-FLIM (Förster resonance energy transfer between two fluorophores detected by fluorescence lifetime imaging microscopy) was used. This method is based on the fact that the energy transfer (FRET) from a donor fluorophore (GFP) to an acceptor fluorophore (mRFP) is only possible when a distance between the two fluorophores is no more than 10 nm. Upon coexpression of GFP- and mRFP-fused proteins, the detection of FRET, which can be measured as a reduced excited-state lifetime of GFP donor fluorophore, indicates a physical interaction between the two fusion proteins [32]. Therefore, leaves of *N. benthamiana* plants were agroinfiltrated for coexpression of GFP-BMB1 (donor) with either BMB2-mRFP (acceptor) or non-fused BMB2 (a control without acceptor) (Supplementary Figure S1), and the GFP fluorescence lifetime was measured in PAMBs (Supplementary Figure S2). GFP-BMB1 was also coexpressed with both BMB2 and mRFP as an additional control, in which the acceptor fluorophore was not fused to BMB2. In the presence of both BMB2-mRFP and GFP-BMB1, the GFP excited-state lifetime measured by FLIM was reduced by 0.9 ns compared to the controls (Figure 1A), corresponding to a FRET efficiency of 36%, thus indicating that BMB1 and BMB2 interact in plant cells.

Previous observations indicated that the mRFP-BMB2 fusion protein is able to induce ER tubule constrictions and to increase the PD SEL like non-fused BMB2 [29], but it is dysfunctional in directing the cell-to-cell transport of GFP-BMB1 [27]. Consistently, the presence of mRFP-BMB2 did not cause so dramatic reduction in the GFP-BMB1 excited-state lifetime as BMB2-mRFP did (Figure 1A). This suggests that mRFP inhibits the BMB2 interaction with BMB1 when fused to the BMB2 N-terminus, implying that the BMB1–BMB2 interaction involves the N-terminus of BMB2. When a similar approach was applied to BMB1, we found that the fluorescence lifetime of BMB1-GFP coexpressed with BMB2-mRFP was reduced by 0.11 ns compared to that in negative controls, showing a FRET efficiency of only 4.5% (Figure 1A). This observation could either indicate that GFP fused to the BMB1 C-terminus interferes with the interaction with BMB2, suggesting involvement of the BMB1 C-terminus in this interaction, or reflect a different, compared to the pair GFP-BMB1/BMB2-mRFP, relative spatial positioning of the two fluorophores suppressing FRET.

The BMB1 and BMB2 proteins were expressed in *E. coli* to test their interaction in vitro by Far-Western assay. To reduce the hydrophobicity of BMB2 for expression in bacteria, the BMB2 gene was modified according to the ‘QTY code’ to replace membrane-embedded helices with hydrophilic ones. This approach has been developed for bacterial expression of highly hydrophobic proteins such as chemokine receptors, which are toxic for bacterial cells in their native form [33]. Importantly, transmembrane proteins modified according to the QTY code and purified form bacterial cells have been experimentally shown to retain their native ability for protein–protein interactions [33,34,35]. The purified BMB1 protein expressed in *E. coli* was loaded onto the protein gel; similarly obtained mouse dihydrofolate reductase (DHFR) was used as a negative control. Following electrophoresis, the proteins were transferred to a membrane and incubated with recombinant BMB2-QTY, which was then detected with BMB2-QTY-specific antibodies. Under these conditions, BMB2-QTY was found to bind BMB1 but not DHFR (Figure 1B), thus providing supportive evidence for the interaction between the BMB1 and BMB2 proteins.

### 3.2. BMB1 Region Involved in Interaction with BMB2

**Figure 2:**
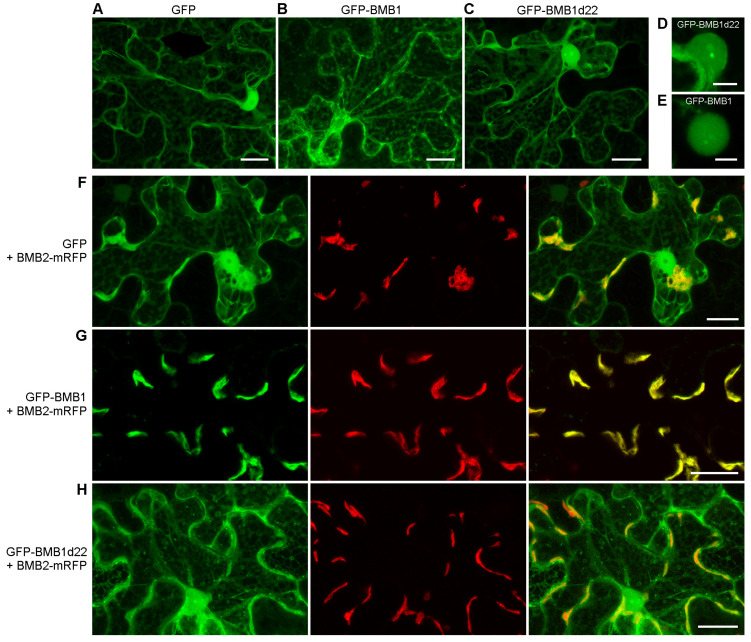
Subcellular localization of GFP-BMB1d22 in comparison to those of GFP and GFP-BMB1. Top line, localization of GFP (**A**), GFP-BMB1 (**B**), GFP-BMB1d22 (**C**) and higher magnification images of nuclei in cells expressing GFP-BMB1d22 (**D**) and GFP-BMB1 (**E**). (**F**), coexpression of GFP and BMB2-mRFP. (**G**), coexpression of GFP-BMB1 and BMB2-mRFP. (**H**), coexpression of GFP-BMB1d22 and BMB2-mRFP. In F-G, left images represent GFP channel, center images—mRFP channel, right images—superposition of images for GFP and mRFP channels. All images are reconstructed from Z-series of optical sections. Scale bar, 20 μm (**A**–**C**,**F**–**H**) and 5 μm (**D**,**E**).

One possible interpretation of the FRET-FLIM data suggests involvement of the BMB1 C-terminal region in the interaction with BMB2. To verify this hypothesis, we generated BMB1d22, a BMB1 mutant with a deletion of 22 C-terminal amino acid residues (Supplementary Figure S1). Confocal microscopy of leaves agroinfiltrated for GFP-BMB1d22 expression revealed that the mutant protein generally retained the subcellular localization typical for GFP-BMB1, being found in both the cytoplasm and the nucleus (Figure 2B,C). Additionally, in nuclei of examined cells, GFP-BMB1d22 concentrated in a structure resembling the nucleolus and, more pronounced, in a small subnuclear body (Figure 2D). Such localization in the nucleus was not previously reported for the wild type (wt) BMB1 protein. Therefore, to verify that BMB1d22 differed from wt BMB1 in its localization to subnuclear structures, the localization of GFP-BMB1 was re-examined. In a fraction of a GFP-BMB1-expressing cells, the subnuclear localization similar to that of BMB1d22, although much less pronounced, was observed, with the small subnuclear body being the main site of GFP-BMB1 concentration in the nucleus (Figure 2E). Hence, the deletion of the 22 C-terminal amino acid residues considerably enhanced the BMB1 association with subnuclear structures, which were barely detectable for the wt protein and therefore overlooked earlier.

To determine the influence of the introduced deletion on BMB1 interaction with BMB2, GFP-BMB1d22 was coexpressed with BMB2-mRFP by agroinfiltration. As controls, GFP-BMB1 and GFP were used. In the presence of BMB2-mRFP, GFP-BMB1 was fully targeted to BMB2-mRFP-containing PAMBs as already demonstrated in previous experiments [27,29]. GFP-BMB1-specific fluorescence was observed neither in the cytoplasm nor in the nucleus (Figure 2G). GFP coexpressed with BMB2-mRFP retained its localization in the cytoplasm and the nucleus. In addition, GFP also associated with PAMBs (Figure 2F), in agreement with the previous observation that PAMBs are situated in cytoplasmic sack regions containing diffusely localized GFP [27]. GFP-BMB1d22 coexpressed with BMB2-mRFP localized to the nucleus and the cytoplasm and also to PAMBs, likely in PAMB-containing cytoplasmic sacks as non-fused GFP (Figure 2H). Therefore, the deletion of BMB1 22 C-terminal amino acid residues resulted in inability of the protein to be relocalized to PAMBs from the cytoplasm and the nucleus in the presence of BMB2, as it is found for the wt BMB1 protein. This inhibition of specific BMB2-dependent BMB1 targeting to PAMBs supports the hypothesis that the BMB1 C-terminal region is involved in interaction with BMB2. To analyze whether this region is sufficient for interaction with BMB2, it was fused to the C-terminus of GFP to give the GFP+22 construct. In cells of agroinfiltrated leaves, GFP+22 localized similarly to GFP, and coexpression with BMB2-mRFP caused no detectable changes in GFP+22 localization (Supplementary Figure S3). These observations may suggest that the 22 C-terminal residues of BMB1 is necessary, but not sufficient for the BMB1 interaction with BMB2.

**Figure 3:**
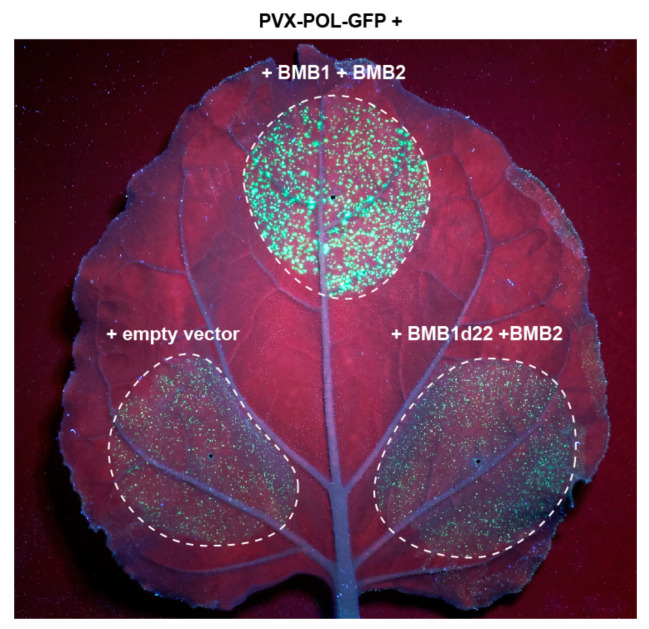
Analysis of functional competence of BMB1d22. Complementation of cell-to-cell movement of PVX-POL-GFP by combinations of BMB1 with BMB2, BMB1d22 and empty vector (negative control) is shown. The leaf was imaged under UV light at 5 d.p.a. Dashed lines encircle infiltrated areas.

To analyze the functional significance of BMB1-BMB2 interaction mediated by the BMB1 C-terminal region, we used a complementation test with PVX-POL-GFP, a *Potato virus X* (PVX) genome-derived reporter construct [36]. PVX-POL-GFP replicates and expresses GFP in initially infected cells but is deficient in cell-to-cell movement as the PVX MP and capsid protein genes are not present in this construct. As reported earlier, infiltration of *N. benthamiana* leaves with highly diluted agrobacterial culture carrying PVX-POL-GFP resulted in individual fluorescent cells, whereas coexpression of PVX-POL-GFP with both BMB1 and BMB2 led to the formation of multicellular infection loci [27] (Figure 3, Supplementary Figure S4) due to complementation of virus transport by the HGSV proteins. When PVX-POL-GFP was coexpressed with BMB1d22 and BMB2, no transport complementation was observed (Figure 3), suggesting that the BMB1 C-terminal region is essential for BMB1 functions in cell-to-cell transport, likely due to involvement of this BMB1 region in the interaction with BMB2.

### 3.3. BMB2 Regions Involved in Interaction with BMB1

**Figure 4:**
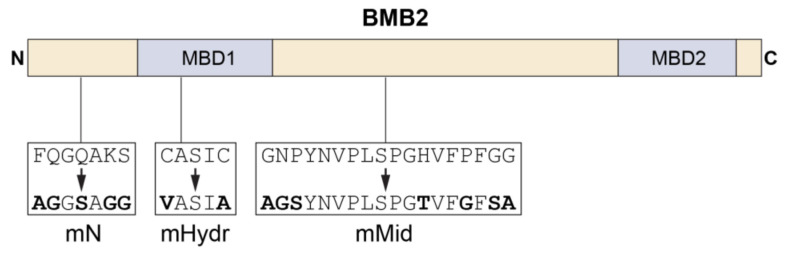
Schematic representation of BMB2 mutants used in this study. The BMB2 sequence is shown as a box with the positions of protein membrane-binding domains (MBD1 and MBD2) indicated. The N-terminal, central and C-terminal hydrophilic regions are shown in yellow color. Boxes below the BMB2 schematic drawing show amino-acid substitutions introduced into the BMB2 sequence to generate mutants BMB2-mN, BMB2-mHydr and BMB2-mMid.

To map BMB2 region(s) involved in the interaction with BMB1, BMB2 was subjected to site-directed mutagenesis. According to previous sequence analysis [28,37], BMB2 has two highly hydrophobic membrane-binding domains (MBDs), which separate the N-terminal hydrophilic region, the central hydrophilic domain, and the protein three hydrophilic C-terminal amino acid residues (Figure 4). Site-directed mutations introduced in the central hydrophilic domain and affecting residues conserved in BMB2 proteins (Supplementary Figure S5) gave the mutant BMB2-mMid (Figure 4). Mutations introduced into the non-conserved N-terminal BMB2 region and affecting charged, polar and aromatic residues that may take part in protein-protein interaction gave mutant BMB2-mN (Figure 4). The mutant BMB2-Hydr (Figure 4) was designed to carry substitutions of two MBD1 cysteine residues, which are also found, although not conserved in their position, in MBD1 of other BMB2 proteins (Supplementary Figure S5) and might affect protein conformation.

**Figure 5:**
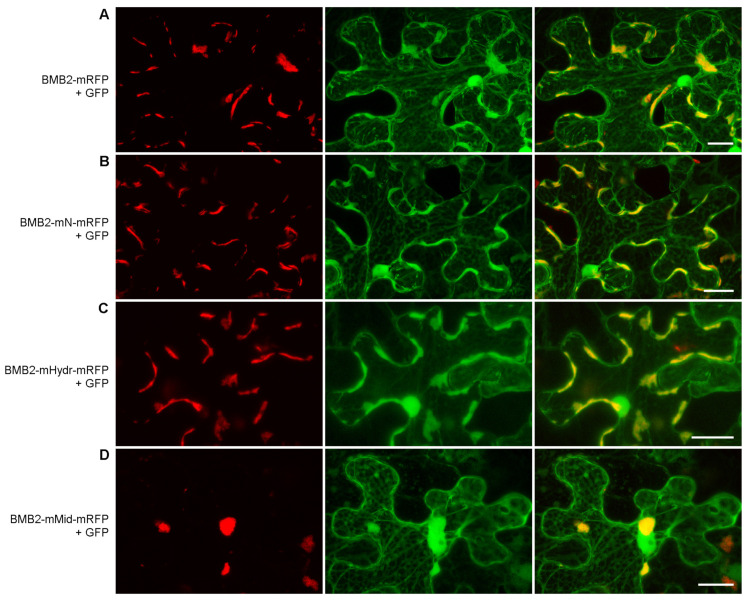
Subcellular localization of BMB2 mutants BMB2-mN (**B**), BMB2-mHydr (**C**) and BMB2-mMid (**D**). The mutants and the wild type protein (**A**) taken as a control were coexpressed with GFP to visualize cells. Left images, mRFP channel. Center images, GFP channel. Right images, superposition of images for GFP and mRFP channels. All images are reconstructed from Z-series of optical sections. Scale bar, 20 μm.

In *N. benthamiana* leaves agroinfiltrated for expression of BMB2 mutants, the subcellular localization of mRFP-fused BMB2-mN and BMB2-Hydr was similar to that of wt BMB2-mRFP (Figure 5A–C), showing that the introduced mutations did not affect the protein targeting to PAMBs. By contrast, BMB2-mMid-mRFP was typically localized to several large abnormal aggregates in the cytoplasm, whereas BMB2-specific PAMBs were not observed for this mutant (Figure 5D), demonstrating that BMB2 aggregation in non-physiological structures could prevent proper protein targeting.

**Figure 6:**
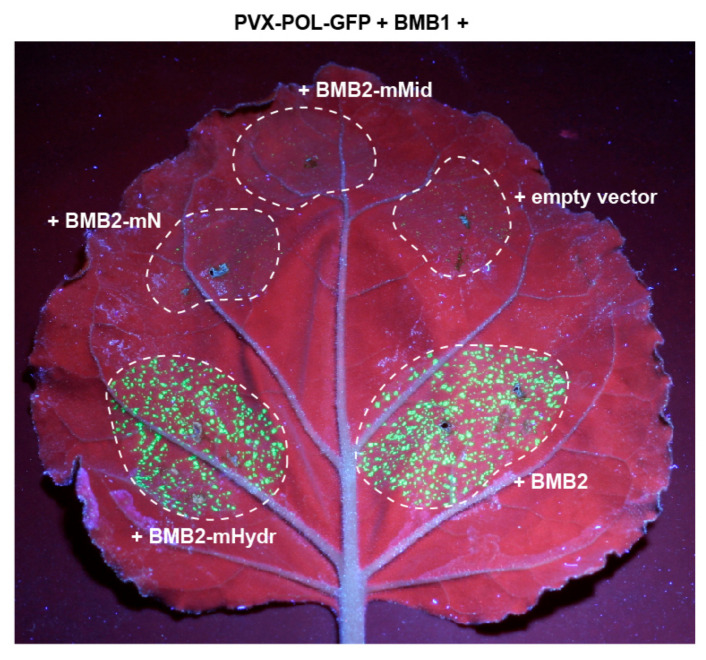
Analysis of functional competence of BMB2 mutants. Complementation of cell-to-cell movement of PVX-POL-GFP by combinations of BMB1 with BMB2 (positive control), BMB2-mN, BMB2-mHydr, BMB2-mMid and empty vector (negative control) is shown. The leaf was imaged under UV light at 5 d.p.a. Dashed lines encircle infiltrated areas.

Next, the functional competence of BMB2 mutants in virus cell-to-cell transport was analyzed in the PVX-POL-GFP complementation test. Leaves were agroinfiltrated for coexpression of PVX-POL-GFP with BMB1 and either wt BMB2, or the individual BMB2 mutants. Of three mutants tested, only BMB2-Hydr retained the functional ability of the wild-type protein in virus transport, whereas BMB2-mN and BMB2-mMid exhibited no complementation of PVX-POL-GFP cell-to-cell movement (Figure 6).

**Figure 7:**
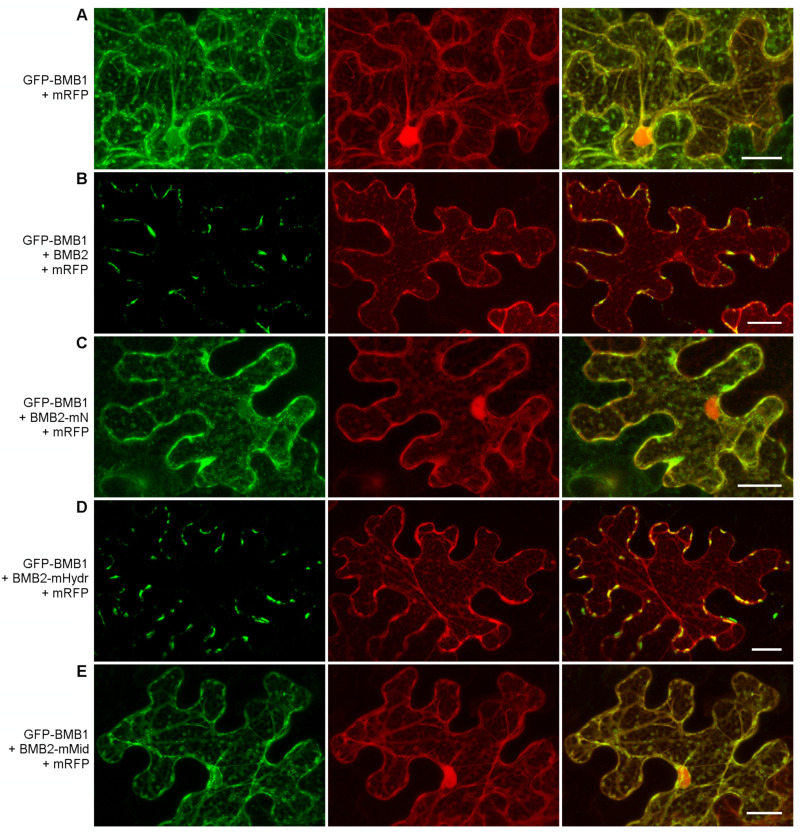
Analysis of the ability of BMB2 mutants to direct GFP-BMB1 to PAMBs. GFP-BMB1 was coexpressed with mRFP (used to visualize the cell shape and the nuclei) and BMB2 mutants (**C**–**E**), as indicated on the left. Coexpression with the wild type BMB2 (**B**) was used as a control. The coexpression of GFP-BMB1 with mRFP (**A**) represented a negative control. Left images, GFP channel. Center images, mRFP channel. Right images, superposition of images for GFP and mRFP channels. Scale bar, 20 μm.

To test whether the BMB2 mutants were capable of directing the intracellular transport of BMB1, GFP-BMB1 was coexpressed with non-fused mutants. Among the mutant BMB2 proteins, only BMB2-Hydr was found to direct BMB1 to PAMBs (Figure 7). Thus, the cysteine residues in the BMB2 MBD1 are neither essential for BMB2 localization to PAMBs and the ability of BMB2 to recruit BMB1 to PAMBs, nor for BMB2 function in virus transport. The failure of BMB2-mMid to recruit GFP-BMB1 to PAMBs (Figure 7E) correlates with its sequestration into aggregates (Figure 5D) and with its inability to support the cell-to-cell transport of the PVX-POL-GFP reporter construct in combination with BMB1 (Figure 6). Likewise, the inability of BMB2-mN to direct GFP-BMB1 to PAMBs (Figure 7C) also is in agreement with the inability of this mutant to function in the PVX-POL-GFP complementation test (Figure 6). BMB2-mN formed typical PAMBs (Figure 5B) and therefore retained part of its functions. However, its inability to target BMB1 to PAMBs suggests the involvement of its N-terminal region in the interaction with BMB1, in agreement with the FRET-FLIM data showing that mRFP fused to the BMB2 N-terminus has no influence on the GFP-BMB1 excited-state lifetime, and therefore suggesting the possibility of interaction between the BMB2 N-terminus and BMB1 (Figure 1A).

### 3.4. Verification of Identified Interaction Regions by FRET-FLIM

**Figure 8:**
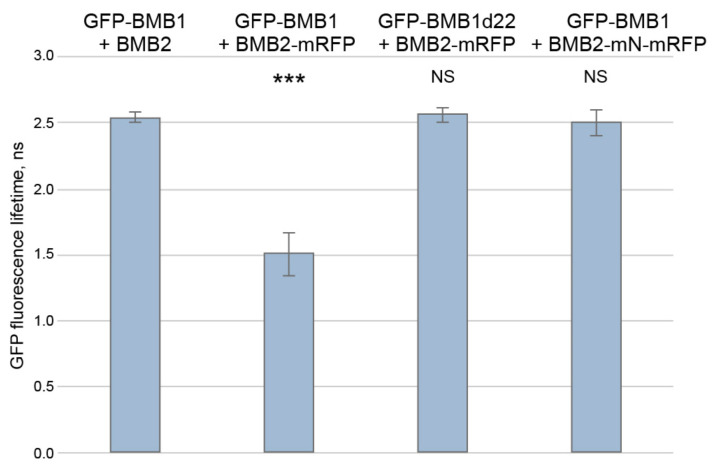
FRET-FLIM analysis of role of the BMB1 C-terminal region and the BMB2 N-terminal region in the interaction between BMB1 and BMB2. Measurements were carried out in cells of *N. benthamiana* leaves agroinfiltrated for expression of the indicated pairs of proteins. The bars represent the average GFP fluorescence lifetimes (ns), error bars indicate the standard deviation. Number of independent measurements (N) was 48 for each tested protein combination. Above the bars, the significance of difference from the data for GFP-BMB1 + BMB2 is indicated; asterisks indicate a statistically significant difference (***, *p* < 0.001) according to a Student’s *t*-test; NS—not statistically significant (*p* ≥ 0.05).

As the FRET-FLIM and mutagenesis data suggested that the BMB1 C-terminal region and the BMB2 N-terminal region could be involved in the interaction of the two proteins, we further verified this conclusion by FRET-FLIM measurements using the respective BMB1 and BMB2 mutants. To this end, leaves of *N. benthamiana* plants were agroinfiltrated for coexpression of GFP-BMB1d22 with BMB2-mRFP and GFP-BMB1 with BMB2-mN-mRFP. As controls, GFP-BMB1 was coexpressed with either BMB2 (a negative control without acceptor), or BMB2-mRFP (a positive control of interacting wt proteins). The GFP fluorescence lifetimes in PAMBs measured for the two protein pairs involving the mutants GFP-BMB1d22 and BMB2-mN-mRFP were similar to that in the negative control, whereas the GFP excited-state lifetime for the interacting wt proteins was significantly reduced (Figure 8). Therefore, the FRET-FLIM data confirm that the interaction between the HGSV BMB proteins involves the BMB1 C-terminal region and the BMB2 N-terminal region.

### 3.5. Mimicking the BMB1-BMB2 Complex

**Figure 9:**
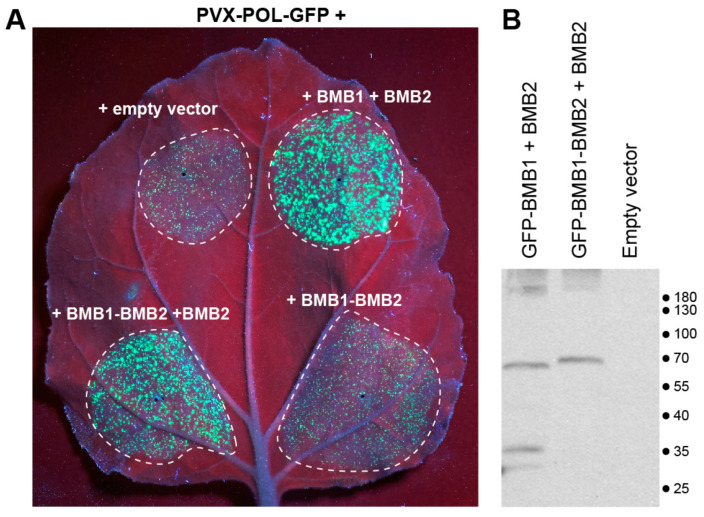
Analysis of properties of the BMB1-BMB2 fusion protein. (**A**). Analysis of functional competence of BMB1-BMB2 in PVX-POL-GFP complementation test. Complementation of cell-to-cell movement of PVX-POL-GFP by combinations of BMB1 with BMB2 (positive control), BMB1-BMB2, BMB1-BMB2 + BMB2 and empty vector (negative control) is shown. The leaf was imaged under UV light at 5 d.p.a. Dashed lines encircle infiltrated areas. (**B**). Western blot analysis of GFP-BMB1-BMB2 with GFP-specific antibodies in *N. benthamiana* leaves agroinfiltrated for coexpression of GFP-BMB1-BMB2 and BMB2. In controls, leaves were agroinfiltrated for expression of either GFP-BMB1 and BMB2, or an empty vector. Positions of molecular weight markers (in kDa) are shown on the right.

As the interaction between the two HGSV MPs involves the BMB1 C-terminal region and the BMB2 N-terminal region, we wondered whether the BMB1:BMB2 complex could be mimicked by the fusion protein BMB1-BMB2, in which BMB2 was covalently linked to the BMB1 C-terminus. However, when the functional competence of BMB1-BMB2 was analyzed in the PVX-POL-GFP complementation test, we found that the BMB1-BMB2 fusion protein was unable to complement the virus cell-to-cell transport (Figure 9A). This observation might indicate that BMB-mediated virus transport requires BMB1 and/or BMB2 as individual polypeptides in addition to the BMB1:BMB2 complex. To test this hypothesis, BMB1:BMB2 was coexpressed with either BMB1 or BMB2 in the PVX-POL-GFP complementation test. Interestingly, BMB2 combined with BMB1-BMB2 restored the complementation of virus transport (Figure 9A), whereas coexpression of BMB1-BMB2 with BMB1 did not have such effect (Supplementary Figure S6). It should be noted, however, that the leaf areas infiltrated for coexpression of BMB1-BMB2 and BMB1 exhibited senescence at the time point of 5 d.p.a., when results of the complementation test are typically recorded, complicating the imaging of such leaves. To rule out the possibility that the BMB1-BMB2 fusion protein is unstable, GFP-fused BMB1-BMB2 expressed in *N. benthamiana* leaves was analyzed by Western blotting with GFP-specific antibodies. GFP-BMB1-BMB2 was detected as a band with the molecular weight of the full-length fusion protein, and no lower-molecular-weight bands were found (Figure 9B). Collectively, these data suggest that the BMB1-BMB2 fusion protein and additional BMB2 protein can cooperate to mediate virus transport, whereas BMB1-BMB2 fusion protein and additional BMB1 cannot.

**Figure 10:**
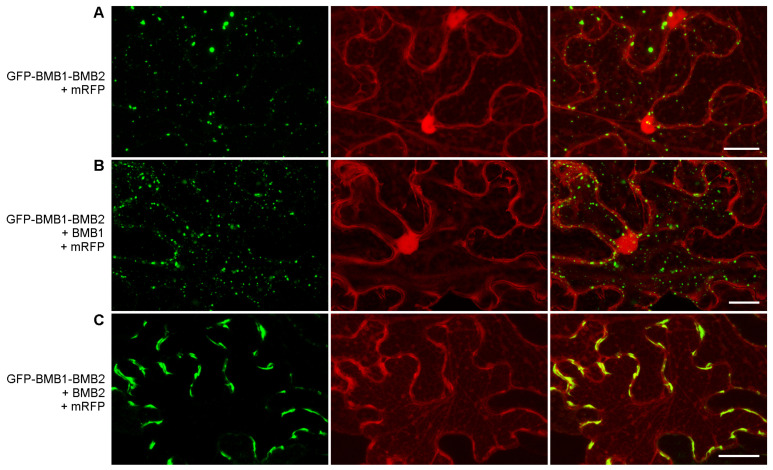
Subcellular localization of the BMB1-BMB2 fusion protein. GFP-BMB1-BMB2 was coexpressed with mRFP used to visualize the cell shape and the nuclei (**A**), or with mRFP and either BMB1 (**B**) or BMB2 (**C**). Left images, GFP channel. Center images, mRFP channel. Right images, superposition of images for GFP and mRFP channels. Scale bar, 20 μm.

Imaging of GFP-BMB1-BMB2 revealed that this fusion protein localized to numerous tiny bodies dispersed throughout the cytoplasm (Figure 10A). This distribution drastically differs from the localization patterns of both BMB1 and BMB2. Upon coexpression with BMB1, GFP-BMB1-BMB2 generally retained its localization in the cytoplasmic bodies, whereas in the presence of BMB2 the GFP-BMB1-BMB2 fusion protein was found in typical BMB2-specific PAMBs (Figure 10B,C). These observations corroborate the complementation data and demonstrate that BMB2 can interact with GFP-BMB1-BMB2 and direct the fusion protein to PAMBs. This targeting can account for the observed complementation of virus transport by the combination of BMB1-BMB2 and BMB2.

## 4. Discussion

The data presented in this paper demonstrate that the BMB2-dependent targeting of BMB1 to BMB2-containing PAMBs requires a specific interaction between the two proteins. This interaction involves the C-terminal BMB1 region and the N-terminal region of BMB2. Whereas the BMB1 localization is mostly cytoplasmic, BMB2 is associated with the ER membranes and is believed to be delivered to PAMBs by translocation along cortical ER tubules [30]. Therefore, we presume that BMB1 interacts with BMB2 upon cotranslational integration of the latter into the ER membrane, allowing BMB1 to be codelivered to PD as a part of a BMB1:BMB2 complex. The interaction of the two proteins early after translation in virus-infected cells likely occurs in VRCs where the synthesis of virus RNAs, including mRNAs for expression of MPs, and their translation take place [1]. As VRCs occur at PD as well as at other cortical sites in epidermal cells [1,8,38], the BMB1:BMB2 complex may form close to the PD entrance.

As BMB1 and BMB2 are encoded in the HGSV genome as individual proteins rather than a single polypeptide, it is likely that the BMB-mediated virus transport requires, in addition to the BMB1:BMB2 complex, the functions of BMB1 and/or BMB2 as individual proteins. As an indirect support of this view, the BMB1-BMB2 fusion protein mechanistically mimicking the BMB1:BMB2 complex is unable to mediate virus transport on its own, but is however functional in the presence of BMB2, but not BMB1. As the functional competence of the covalent BMB1-BMB2 fusion protein could be different from that of BMB1:BMB2 complex, it is currently unclear whether the individual BMB2 polypeptide is required for virus transport under natural conditions. Thus, further studies are required to determine the functions of individual BMB1 and BMB2 proteins, as well as their complex, in virus cell-to-cell movement.

Earlier, we showed that the fusion of mRFP to the N- and C-terminus of BMB2 has different effects on BMB2 function. The mRFP-BMB2 fusion protein localizes to PAMBs and the PD interior, induces constrictions of the ER tubules, and increases the PD SEL. By contrast, BMB2-mRFP, although capable of localization to PAMBs, fails to induce ER constrictions, to localize to PD, and to increase the PD SEL [29]. However, both BMB2 fusions are dysfunctional in mediating viral and BMB1 transport through PD [27]. The data presented here indicate that the dysfunction of mRFP-BMB2 in virus movement is caused by the inability of mRFP-BMB2 to interact with BMB1, while BMB2-mRFP retains the ability to interact with BMB1 but is deficient in protein essential functions that likely result from BMB2 conformational changes caused by mRFP fused to the protein C-terminus.

The central region located between two hydrophobic membrane-associated domains is conserved in BMB2 proteins and has a distant relation to the corresponding region of TGB2 proteins [28,37]. Mutations introduced in this region result in protein aggregation in large cytoplasmic inclusions that prevents correct BMB2 targeting to PAMBs. The mutant protein sequestered in large inclusions is unable to interact with BMB1 and direct the latter to the inclusions. However, these observations do not exclude the possibility that the central regions of the wt BMB2 protein could still function, in addition to the N-terminal region, in the interaction with BMB1. Such interaction is possible as the BMB2 central region, as well as the N-terminal region, is located in the cytoplasm [29]. Interestingly, mutations introduced in the central region of *Potato mop-top virus* TGB2 protein have been shown to interfere with the ability of TGB2 to target the TGB1 protein to PAMBs in the presence of TGB3. However, it remains unresolved whether this effect resulted from TGB2 misfolding/mislocalization or impaired TGB1 binding per se [19]. Therefore, the potential role of the BMB2/TGB2 central region in interaction with BMB1/TGB1 is a subject of further investigations.

## 5. Conclusions

Understanding the interaction between MPs, as well as the specific functions of individual proteins, is of key importance for unraveling the molecular mechanism of plant virus cell-to-cell movement. The specific interaction of BMB1 and BMB2 proteins uncovered in this paper can open new perspectives in studies of virus transport mechanisms.

## Data Availability

Not applicable.

## Supplementary Materials

The following supporting information can be downloaded at: https://www.mdpi.com/article/10.3390/v14122742/s1, Figure S1: Schematic representation of HGSV-specific constructs used in this study; Figure S2: Analysis of interaction between BMB1 and BMB2 by FRET-FLIM; Figure S3: Subcellular localization of GFP+22; Figure S4: Complementation of PVX-POL-GFP cell-to-cell transport. Figure S5: Amino acid sequence alignment of BMB2 proteins; Figure S6: Complementation of cell-to-cell movement of PVX-POL-GFP by BMB1-BMB2 and BMB1-BMB2 combination with BMB1. Table S1: Primers used in this study; Table S2: Synthetic genes used in this study.

## References

[1] Heinlein M. (2015). Plant Virus Replication and Movement. Virology.

[2] Heinlein M. (2015). Plasmodesmata: Channels for Viruses on the Move. Methods Mol. Biol..

[3] Li Z.P., Paterlini A., Glavier M., Bayer E.M. (2021). Intercellular Trafficking via Plasmodesmata: Molecular Layers of Complexity. Cell Mol. Life Sci..

[4] Reagan B.C., Burch-Smith T.M. (2020). Viruses Reveal the Secrets of Plasmodesmal Cell Biology. Mol. Plant-Microbe Interact..

[5] Huang C., Heinlein M. (2022). Function of Plasmodesmata in the Interaction of Plants with Microbes and Viruses. Methods Mol. Biol..

[6] Tilsner J., Taliansky M.E., Torrance L. (2014). Plant Virus Movement.

[7] Lucas W.J. (2006). Plant Viral Movement Proteins: Agents for Cell-to-Cell Trafficking of Viral Genomes. Virology.

[8] Wu X., Cheng X. (2020). Intercellular Movement of Plant RNA Viruses: Targeting Replication Complexes to the Plasmodesma for Both Accuracy and Efficiency. Traffic.

[9] Peña E.J., Robles Luna G., Heinlein M. (2021). In Vivo Imaging of Tagged MRNA in Plant Tissues Using the Bacterial Transcriptional Antiterminator BglG. Plant J..

[10] Solovyev A.G., Atabekova A.K., Lezzhov A.A., Solovieva A.D., Chergintsev D.A., Morozov S.Y. (2022). Distinct Mechanisms of Endomembrane Reorganization Determine Dissimilar Transport Pathways in Plant RNA Viruses. Plants.

[11] Citovsky V. (1999). Tobacco Mosaic Virus: A Pioneer to Cell–to–Cell Movement. Philos. Trans. R. Soc. London..

[12] Morozov S.Y., Solovyev A.G. (2003). Triple Gene Block: Modular Design of a Multifunctional Machine for Plant Virus Movement. J. Gen. Virol..

[13] Wu C.-H., Lee S.-C., Wang C.-W. (2011). Viral Protein Targeting to the Cortical Endoplasmic Reticulum Is Required for Cell-Cell Spreading in Plants. J. Cell Biol..

[14] Lee S.-C., Wu C.-H., Wang C.-W. (2010). Traffic of a Viral Movement Protein Complex to the Highly Curved Tubules of the Cortical Endoplasmic Reticulum. Traffic.

[15] Solovyev A.G., Stroganova T.A., Zamyatnin A.A., Fedorkin O.N., Schiemann J., Morozov S.Y. (2000). Subcellular Sorting of Small Membrane-Associated Triple Gene Block Proteins: TGBp3-Assisted Targeting of TGBp2. Virology.

[16] Schepetilnikov M.V., Manske U., Solovyev A.G., Zamyatnin A.A., Schiemann J., Morozov S.Y. (2005). The Hydrophobic Segment of Potato Virus X TGBp3 Is a Major Determinant of the Protein Intracellular Trafficking. J. Gen. Virol..

[17] Tilsner J., Cowan G.H., Roberts A.G., Chapman S.N., Ziegler A., Savenkov E., Torrance L. (2010). Plasmodesmal Targeting and Intercellular Movement of Potato Mop-Top Pomovirus Is Mediated by a Membrane Anchored Tyrosine-Based Motif on the Lumenal Side of the Endoplasmic Reticulum and the C-Terminal Transmembrane Domain in the TGB3 Movement Protein. Virology.

[18] Schepetilnikov M.V., Solovyev A.G., Gorshkova E.N., Schiemann J., Prokhnevsky A.I., Dolja V.V., Morozov S.Y. (2008). Intracellular Targeting of a Hordeiviral Membrane-Spanning Movement Protein: Sequence Requirements and Involvement of an Unconventional Mechanism. J. Virol..

[19] Zamyatnin A.A., Solovyev A.G., Savenkov E.I., Germundsson A., Sandgren M., Valkonen J.P.T., Morozov S.Y. (2004). Transient Coexpression of Individual Genes Encoded by the Triple Gene Block of Potato Mop-Top Virus Reveals Requirements for TGBp1 Trafficking. Mol. Plant-Microbe Interact..

[20] Shemyakina E.A., Solovyev A.G., Leonova O.G., Popenko V.I., Schiemann J., Morozov S.Y. (2011). The Role of Microtubule Association in Plasmodesmal Targeting of Potato Mop-Top Virus Movement Protein TGBp1. Open Virol. J..

[21] Chou Y.L., Hung Y.J., Tseng Y.H., Hsu H.T., Yang J.Y., Wung C.H., Lin N.S., Meng M., Hsu Y.H., Chang B.Y. (2013). The Stable Association of Virion with the Triple-Gene-Block Protein 3-Based Complex of Bamboo Mosaic Virus. PLoS Pathog..

[22] Lim H.-S., Bragg J.N., Ganesan U., Lawrence D.M., Yu J., Isogai M., Hammond J., Jackson A.O. (2008). Triple Gene Block Protein Interactions Involved in Movement of Barley Stripe Mosaic Virus. J. Virol..

[23] Ho T.-L., Lee H.-C., Chou Y.-L., Tseng Y.-H., Huang W.-C., Wung C.-H., Lin N.-S., Hsu Y.-H., Chang B.-Y. (2017). The Cysteine Residues at the C-Terminal Tail of Bamboo Mosaic Virus Triple Gene Block Protein 2 Are Critical for Efficient Plasmodesmata Localization of Protein 1 in the Same Block. Virology.

[24] Park M.-R., Jeong R.-D., Kim K.-H. (2014). Understanding the Intracellular Trafficking and Intercellular Transport of Potexviruses in Their Host Plants. Front. Plant Sci..

[25] Melzer M.J., Sether D.M., Borth W.B., Hu J.S. (2012). Characterization of a Virus Infecting Citrus Volkameriana with Citrus Leprosis-like Symptoms. Phytopathology.

[26] Morozov S.Y.S.Y., Solovyev A.G.A.G. (2015). Phylogenetic Relationship of Some “Accessory” Helicases of Plant Positive-Stranded RNA Viruses: Toward Understanding the Evolution of Triple Gene Block. Front. Microbiol..

[27] Lazareva E.A., Lezzhov A.A., Komarova T.V., Morozov S.Y., Heinlein M., Solovyev A.G. (2017). A Novel Block of Plant Virus Movement Genes. Mol. Plant Pathol..

[28] Morozov S.Y., Solovyev A.G. (2012). Did Silencing Suppression Counter-Defensive Strategy Contribute to Origin and Evolution of the Triple Gene Block Coding for Plant Virus Movement Proteins?. Front. Plant Sci..

[29] Lazareva E.A.A., Lezzhov A.A.A., Chergintsev D.A.A., Golyshev S.A.A., Dolja V.V.V., Morozov S.Y.Y., Heinlein M., Solovyev A.G.G. (2021). Reticulon-like Properties of a Plant Virus-Encoded Movement Protein. New Phytol..

[30] Lazareva E.A., Lezzhov A.A., Golyshev S.A., Morozov S.Y., Heinlein M., Solovyev A.G. (2017). Similarities in Intracellular Transport of Plant Viral Movement Proteins BMB2 and TGB3. J. Gen. Virol..

[31] Solovyev A.G., Minina E.A., Makarova S.S., Erokhina T.N., Makarov V.V., Kaplan I.B., Kopertekh L., Schiemann J., Richert-Pöggeler K.R., Morozov S.Y. (2013). Subcellular Localization and Self-Interaction of Plant-Specific Nt-4/1 Protein. Biochimie.

[32] Schoberer J., Botchway S.W. (2014). Investigating Protein–Protein Interactions in the Plant Endomembrane System Using Multiphoton-Induced FRET-FLIM. Methods Mol. Biol..

[33] Zhang S., Tao F., Qing R., Tang H., Skuhersky M., Corin K., Tegler L., Wassie A., Wassie B., Kwon Y. (2018). QTY Code Enables Design of Detergent-Free Chemokine Receptors That Retain Ligand-Binding Activities. Proc. Natl. Acad. Sci. USA.

[34] Tegler L., Corin K., Pick H., Brookes J., Skuhersky M., Vogel H., Zhang S. (2020). The G Protein Coupled Receptor CXCR4 Designed by the QTY Code Becomes More Hydrophilic and Retains Cell Signaling Activity. Sci. Rep..

[35] Hao S., Jin D., Zhang S., Qing R. (2020). QTY Code-Designed Water-Soluble Fc-Fusion Cytokine Receptors Bind to Their Respective Ligands. QRB Discov..

[36] Lazareva E.A., Atabekova A.K., Lezzhov A.A., Morozov S.Y., Heinlein M., Solovyev A.G. (2022). Virus Genome-Based Reporter for Analyzing Viral Movement Proteins and Plasmodesmata Permeability. Methods Mol. Biol..

[37] Solovyev A.G., Morozov S.Y. (2017). Non-Replicative Integral Membrane Proteins Encoded by Plant Alpha-like Viruses: Emergence of Diverse Orphan ORFs and Movement Protein Genes. Front. Plant Sci..

[38] Tilsner J., Linnik O., Louveaux M., Roberts I.M., Chapman S.N., Oparka K.J. (2013). Replication and Trafficking of a Plant Virus Are Coupled at the Entrances of Plasmodesmata. J. Cell Biol..

