# Supplementary material for: Interaction between Movement Proteins of Hibiscus green spot virus
Source: Viruses. 2022 Dec 8;14(12):2742. doi: 10.3390/v14122742 (PMC9780815; doi:10.3390/v14122742)
Supplement: Supplementary file 1 [file viruses-14-02742-s001.zip › viruses-2016007-supplementary.pdf]

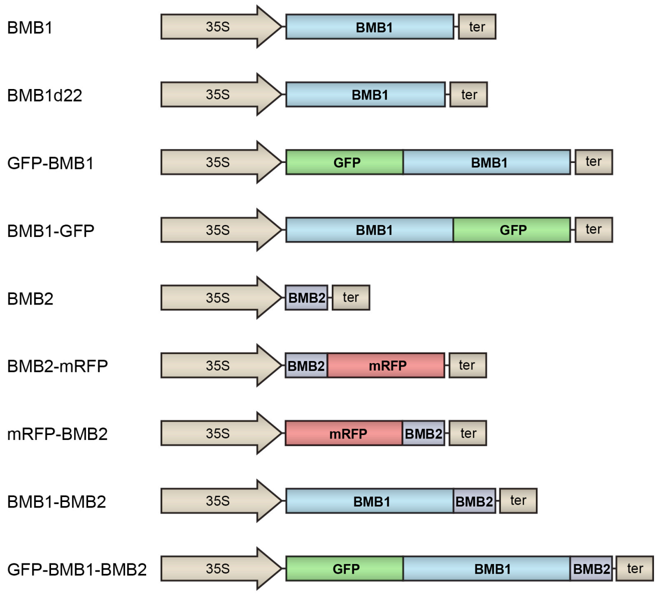

Figure S1. Schematic representation of HGSV-specific constructs used in this study. Expression cassettes of binary vectors are presented. Genes are shown as boxes. 35S, the *Cauliflower mosaic virus* 35S promoter; ter, *Cauliflower mosaic virus* 35S terminator.

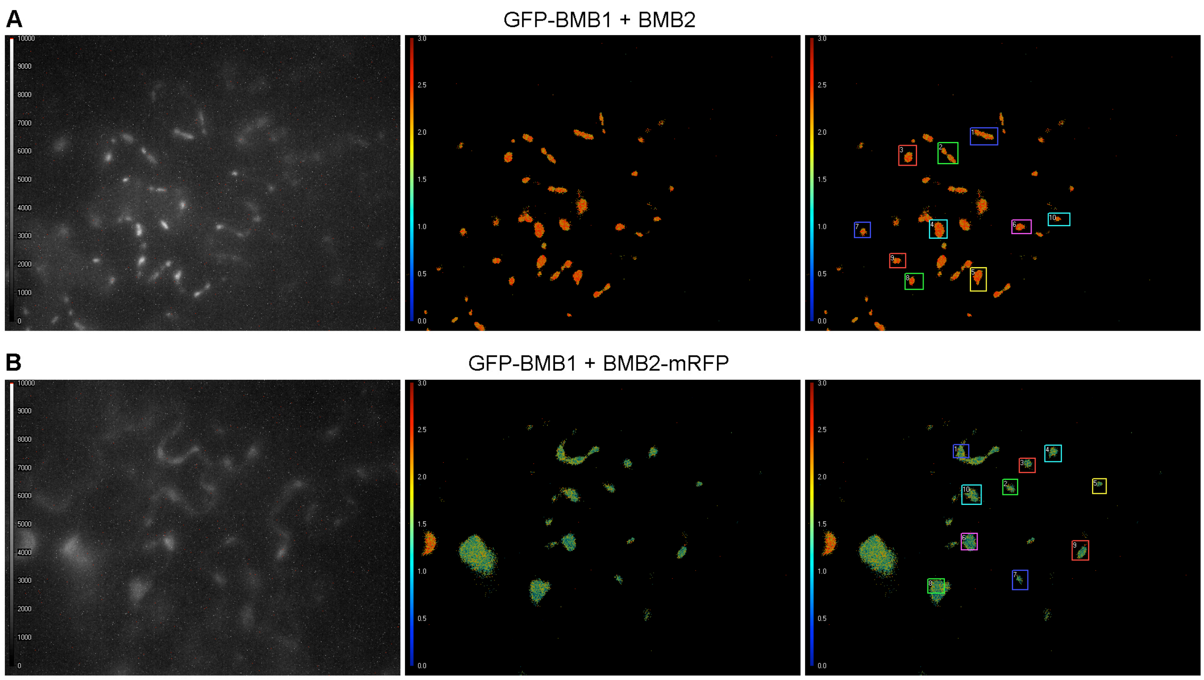

Figure S2. Analysis of interaction between BMB1 and BMB2 by FRET-FLIM. Representative FRET-FLIM images of cells coexpressing either the GFP-BMB1 donor with BMB2 (a no-acceptor control) (A), or the GFP-BMB1 donor with the BMB2-mRFP acceptor (B) are shown. Left, donor fluorescence intensity. Center, donor excited-state lifetime map; the color bar indicates lifetime in ns. Right, PAMB-containing areas of measurement shown on the lifetime map.

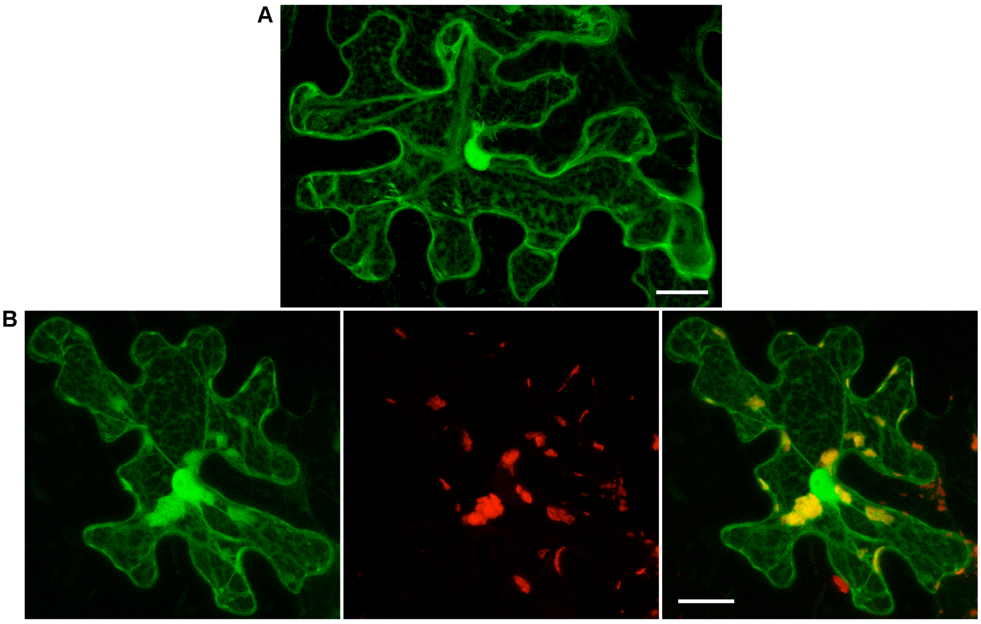

Figure S3. Subcellular localization of GFP+22. Confocal microscopy of cells either expressing GFP+22 (A), or coexpressing GFP+22 and BMB2-mRFP (B). Scale bar, 20 μm.

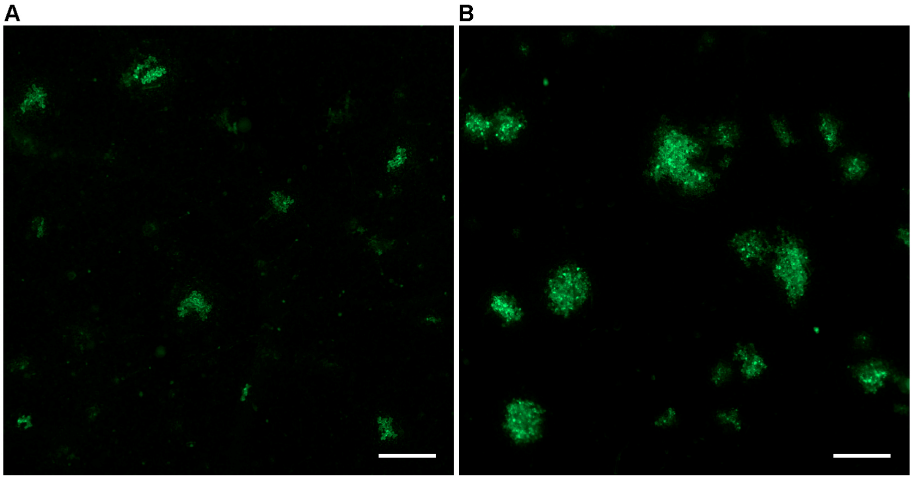

Figure S4. Complementation of PVX-POL-GFP cell-to-cell transport. Representative images showing individual fluorescent cells (A) observed in the absence of cell-to-cell transport in a leaf agroinfiltrated for expression of PVX-POL-GFP and multicellular fluorescent loci (B) observed upon complementation of PVX-POL-GFP cell-to-cell transport in the presence of HGSV MPs. Scale bar, 500 μm.

```
                      mN          mHydr                     mMid
                    ** * **       *   *              ***        *  * **
HGSV          ---MPIFQGQAKSNAPVLFVCASICVIAYFFF-----NPGNPYNVPLSPGHVFPFGGEYA
PisVX         ----MPRFVDNSASSKLILVLCFCMLVSAVYIMY---HPGNPYSVPMAPGHVFPFGGRYA
L.sativus     --MVLVRTPDKKDDTKVIIVYGFMVLIGCCFFIYNSVNPGNPYNVGLAPGHVFPHGGRLI
L.chinensis   MAILNAPGNNVSLNLNIVCVFCCVCLCFICWCM---NNPGNPYDVGFANGFVFPNGGRYM
Q.castanea    ----MPIISNTQGKGSLGLIVCVLVVGFCFWCVC---HPGNPFNVGISPGHVFPHGGSYE

HGSV          VHARFNGPSFPLS--MGGSSVGLLMLCVILLVVVTHM---------
PisVX         ITAEFYPPRGTVW--RDSSNLMVLFICLTICCYLRFR---------
L.sativus     VTAEYNSPPGWFQ-FKECPSLNFLLMFVMALITVIFFSSPRLENNR
L.chinensis   VQAEFLPPKFSLFNFRGNGSFSLVLICVGILVYLRF----------
Q.castanea    IKATFLPQQRSFY-FRESPGLFLLFLCVFLAFVVSKLAI-------
```

Figure S5. Amino acid sequence alignment of BMB2 proteins. The alignment includes proteins encoded by HGSV, *Pistachio virus X* (PisVX), and virus-like RNAs identified in transcriptomes of *Lathyrus sativus*, *Litchi chinensis* and *Quercus castanea*. Membrane-associated sequence segments predicted by the TMHMM algorithm (https://services.healthtech.dtu.dk/service.php?TMHMM-2.0) are shown in yellow color. Residues conserved in the central hydrophilic region are indicated by gray shading. Cysteine residues in the first membrane-binding hydrophobic region are shown in blue color. Residues affected by mutations in mutants mN, mHydr and mMid are shown by asterisks.

PVX-POL-GFP +

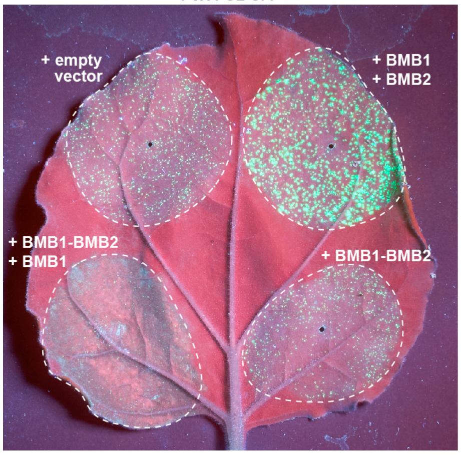

Figure S6. Complementation of cell-to-cell movement of PVX-POL-GFP by BMB1-BMB2 and BMB1-BMB2 combination with BMB1. The leaf was coinfiltrated for expression of PVX-POL-GFP and the following constructs as indicated, empty vector (negative control), BMB1 + BMB2 (positive control), BMB1-BMB2, and BMB1-BMB2 + BMB1. The leaf was imaged under UV light at 5 d.p.a. Dashed lines encircle infiltrated areas.

Table S1. Primers used in this study.

BMB2-mN-P	GCCTCGAGACCATGCCAATTGCTGGAGGTTCAGCGGGGGGTAACGCGCCCGTGT<br>TGTTT
BMB2-mHydr-P	CGCTCGAGACCATGCCGATATTTCAAGGTCAAGCGAAGAGTAACGCGCCCGTGTT<br>GTTTGTGGTTGCTAGCATAGCTGTTATAGCG
BMB2-mMid-ovl-P	TATAATGTACCTTTGAGTCCTGGTACCGTGTTCGGTTTCAGCGCAGAGTACGC
BMB2-mMid-ovl-M	ACCAGGACTCAAAGGTACATTATAAGAACCTGCAGGATTAAAGAAG
BMB1C-BglII-P	GTCCTGATGTTGAGTTTGTGCG
BMB1C-d22-M	CGCTCTAGATTACTGGAAATTATCATGGTCTACCAC
BMB2-C22-P	GCGGATCCCCGGCATATGAAGATTCTCTT
Right	CCTTATCTGGGAACTACTCACACATT
Left	TATCCTTCGCAAGACCCTTCCTCT

Table S2. Synthetic genes used in this study.

Gene	Sequence
BMB1	GGATCCGATGGAAAGCTTCAACTACGTTACCTCTGCGGATCTGGTTTGCTCCCTGTCTCGTTTTCG<br>TGACTTCTACAAGGAACGTAGCGCGTCTCTGTCTGACGACGACGTTTACGCGGACACCACCCGTGA<br>ATACTCTGCTGCCCTGGACAGCACCGAATTCGTAGATTACAACTTCCAGATCTTTCTGCTGAACGG<br>TGTGCCAGGTGGCGGTAAAACCAAATTCGTCTACGAAAACGTTGAAGTGCGTAACTCTTGCGTTGT<br>TGTTCCGTTTAAAGCTCTGAAAGACGAGTACCGCAAACGTGGTTACCGTTCCTTCACCCAGTTCCG<br>TGCACTGCCGTCCGTTCGTTCCGCAGACATCCTGGTAATCGATGAATACACCTGCGTGTGCTACTC<br>TGTTCTGGTGAGCCTGGTTTACAAACTGCGTCCGGCAACCGTTGCCCTGATCGGTGACTTTAATCA<br>GTGCTGGATTCGTGACGGCGAGGGTTTCTCTATGGAACCGTTTGTAAGCACCCTGGTGGTGAACAA<br>GTACCTGTCCGTTTGCTACCGCTGTCCGTGTCCGGACGTAGAGTTTGTTCGTAACTACCTGGACCT<br>GGACATTACCCCGGGCAAATGCGGCTCCACCTGTAGCTGCACCGGCTTCAACGTAGAAACCCTGGT<br>TGAAGGTATGACCTTCGATTACCGCGGTGAAGTTGCGCTGGTGTTCAGCGGCGCGAGCAAATCCTA<br>CCTGGAGTCTATCGGCGTTTCCGCTGTAACCGTCCGTAGCTTCATGGGTAAGCAGGCAGATACCGT<br>TGCGCTGTTCATCCTGAAAGATCACGACATCCGTCTGCTGGGCGTTTCTTCTCTGAAGCTGGTAGC<br>ATTTACCCGTCACGTTAGCAAACTGGTTGTTTACAGCGATATGTCCCACGATGATGTGGTCGCTCT<br>GATCGAAGGTGCGACTGAGTCTGGCGAAGTGGTTGACCACGATAACTTCCAGCCGGCGTACGAGGA<br>CAGCCTGGAACAGTACTTCGACCGCCTGCTGCGTGGCGACTTCTACGTTCTGTAACTCGAG
BMB2-QTY	CTCGAGACCATGCCGATATTTCAAGGTCAAGCGAAGAGTAACGCGCCCACTCAATATACATGTGCT<br>AGTACATGTACTACAGCGTATTACTACTATAATCCTGGTAATCCTTATAATGTACCTTTGAGTCCT<br>GGTCACGTGTTCCCTTTCGGGGGAGAGTACGCGGTTCATGCTAGGTTCAATGGACCGAGTTTCCCT<br>CTTAGTATGGGTGGTTCTAGTACAGGACAGCAAATGCAGTGTACTACACAACAAACTACTACTACG<br>CATATGTAATCTAGA
